# Tissue Inhibitor of Metalloproteinase-3 (TIMP-3) Regulates Hematopoiesis and Bone Formation In Vivo

**DOI:** 10.1371/journal.pone.0013086

**Published:** 2010-09-30

**Authors:** Yi Shen, Ingrid G. Winkler, Valerie Barbier, Natalie A. Sims, Jean Hendy, Jean-Pierre Lévesque

**Affiliations:** 1 Hematopoietic Stem Cell Laboratory, Mater Medical Research Institute, South Brisbane, Queensland, Australia; 2 School of Medicine, University of Queensland, Brisbane, Queensland, Australia; 3 St. Vincent's Institute, Fitzroy, Victoria, Australia; The Ohio State University Medical Center, United States of America

## Abstract

**Background:**

Tissue inhibitor of metalloproteinases-3 (**TIMP-3**) inhibits matrix metalloproteinases and membrane-bound sheddases. TIMP-3 is associated with the extracellular matrix and is expressed in highly remodeling tissues. TIMP-3 function in the hematopoietic system is unknown.

**Methodology/Principal Findings:**

We now report that TIMP-3 is highly expressed in the endosteal region of the bone marrow (**BM**), particularly by osteoblasts, endothelial and multipotent mesenchymal stromal cells which are all important cellular components of hematopoietic stem cell (**HSC**) niches, whereas its expression is very low in mature leukocytes and hematopoietic stem and progenitor cells. A possible role of TIMP-3 as an important niche component was further suggested by its down-regulation during granulocyte colony-stimulating factor-induced mobilization. To further investigate TIMP-3 function, mouse HSC were retrovirally transduced with human TIMP-3 and transplanted into lethally irradiated recipients. TIMP-3 overexpression resulted in decreased frequency of B and T lymphocytes and increased frequency of myeloid cells in blood and BM, increased Lineage-negative Sca-1^+^KIT^+^ cell proliferation *in vivo* and *in vitro* and increased colony-forming cell trafficking to blood and spleen. Finally, over-expression of human TIMP-3 caused a late onset fatal osteosclerosis.

**Conclusions/Significance:**

Our results suggest that TIMP-3 regulates HSC proliferation, differentiation and trafficking in vivo, as well as bone and bone turn-over, and that TIMP-3 is expressed by stromal cells forming HSC niches within the BM. Thus, TIMP-3 may be an important HSC niche component regulating both hematopoiesis and bone remodeling.

## Introduction

Hematopoietic stem cells (**HSC**) have the capacity to self-renew life-long and to generate all blood lineages, while hematopoietic progenitor cells (**HPC**) possess limited differentiation capacity. Hematopoietic stem and progenitor cells (**HSPC**) reside in specific micro-domains of the bone marrow (**BM**) called niches. The molecular composition of niches provides the extrinsic cues that control HSPC fate, such as quiescence, self-renewal or differentiation. Two types of HSC niches have been reported: 1) endosteal or osteoblastic niches where HSC are within 2 cell diameters from osteoblasts lining the endosteum [1]; [2]; [3]; [4]; [5], and 2) vascular niches where HSC are in direct contact with the abluminal side of endothelial cells forming BM sinuses [6]; [7]; [8]. The molecular and cellular composition and function of these HSC niches is under intense investigation. Soluble chemokines (CXCL12/SDF1), soluble cytokines (angiopoietin-1), transmembrane cytokines (KIT ligand, Jagged-1) and cell adhesion molecules (VCAM-1, osteopontin, cadherins, annexin II) are expressed within these niches and control HSC fate [9]; [10]; [11]; [12].

Proteases produced by BM myeloid cells are important regulators of some of these HSC niche components, particularly in stress situations, such as following granulocyte colony-stimulating factor (**G-CSF**) stimulation or during recovery from cytotoxic or radiological insults. The two neutrophil serine-proteases neutrophil elastase and cathepsin G can cleave and inactivate molecules important in HSPC retention within the BM, such as VCAM-1, c-KIT, CXCR4 and CXCL12 [13]; [14]; [15]; [16]; [17]; [18]. Once these interactions are disrupted, HSPC can dislodge from their niches to circulate in the blood. The matrix metalloproteinases (**MMP**)-9 and MT1-MMP (MMP-14) have been reported to play an important role by cleaving and inactivating CXCL12 and KIT ligand during mobilization and recovery from cytotoxic insult [15]; [19]; [20]; [21]. Cysteine protease cathepsin K, produced by osteoclasts, has been reported to cleave CXCL12 and KIT ligand during HSPC mobilization [22]. Therefore proteases produced within the BM regulate the molecular composition of niches.

The activity of proteases is controlled by naturally occurring protease inhibitors. Serine protease inhibitors α1-antitrypsin (serpina1) and α1-antichymotrypsin (serpina3) are expressed in the BM to prevent the accidental cleavage of niche components by neutrophil elastase and cathepsin G [23]; [24]. However during G-CSF- and chemotherapy-induced mobilization, these serpins are down-regulated enabling accumulation of active serine proteases and subsequent cleavage and inactivation of molecules important in HSPC retention within the BM [23]; [25]. Another potentially important family of protease inhibitors is the tissue inhibitors of matrix metalloproteinases (**TIMP**) which comprises 4 members, TIMP-1 to -4. The archetypal TIMP-1 and TIMP-2 possess an erythroid potentiating activity *in vitro* independently of their MMP inhibiting activity [26]; [27]; [28]. However deletion of the TIMP-1 gene or over-expression of TIMP-1 or TIMP-2 *in vivo* did not alter hematopoiesis in steady-state, nor HSPC mobilization in response to G-CSF or cytotoxic injury [29].

Among the TIMP family members, TIMP-3 displays unique molecular features and properties. TIMP-3 has unique domains that interact with extracellular matrix (ECM) components and, unlike the other TIMPs, is mainly bound to tissue ECM [30]; [31]; [32]. It is the only TIMP capable of inhibiting membrane bound MMP, transmembrane MMP and sheddases such as TNF-α converting enzyme (TACE) also called a disintegrin and metalloproteinase (**ADAM**)-17 [33]. Finally, TIMP-3 is a unique competitive inhibitor of vascular endothelial cell growth factor (VEGF)-A binding to VEGF receptor 2 (**VEGF-R2**) [34] and of angiopoietin-1 to its receptor Tie2 [35].

We now report that TIMP-3 is expressed at the endosteum in mouse BM by osteoblastic cells, multipotent mesenchymal stromal cells (**MSC**) and endothelial cells and is the only TIMP down-regulated during G-CSF-induced mobilization. Finally, over-expression of huTIMP-3 in hematopoietic cells results in increased HSPC proliferation and trafficking, and myelopoiesis *in vivo* leading to progressive and ultimately fatal osteosclerosis.

## Results

### Decrease in TIMP-3 Expression during G-CSF Induced Mobilization of HSPC

**Figure 1:**
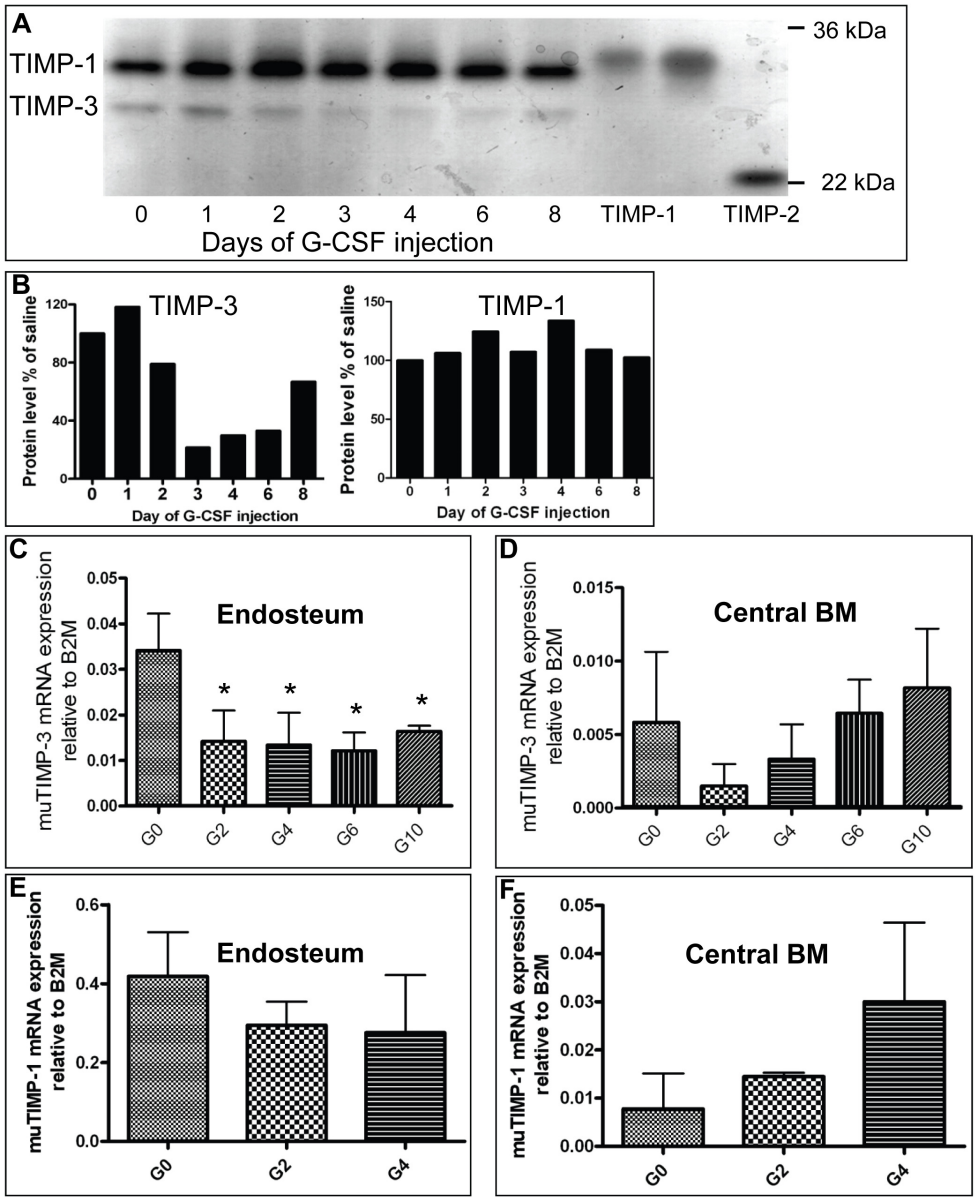
Decreased TIMP-3 expression level during G-CSF induced mobilization. (A) Detection by reverse zymography of TIMP-1 and TIMP-3 proteins from mouse BM fluids following mobilization with G-CSF. G-CSF was administered twice daily from day 0 to day 6 and the left to rest for 2 days (day 8 group). Each sample is a pool of BM fluids from 4 mice per time point. Controls were made with recombinant human TIMP-1 (0.1 and 0.3 ng) and TIMP-2 (1 ng). (B) Quantification of TIMP-3 and TIMP-1 protein concentrations during G-CSF induced mobilization from panel A. (C–D) TIMP-3 mRNA expression by RT-qPCR in cells from the endosteal region (C) and central BM (D). Data are normalized to B2M expression and are average ± SD of at least 4 mice per time-point. * indicates a p value of 0.03 compared to control mice. (E–F) TIMP-1 mRNA expression in endosteal (E) and central BM (F).

Similarly to neutrophil serine-proteases, MMP-9 protein levels increase in the BM during G-CSF-induced mobilization [14]; [18]; [19]. As serpin expression decreases during mobilization [23]; [25], we investigated expression of MMP inhibitors in the mobilized BM. Reverse zymography of pooled BM fluids from G-CSF-mobilized mice revealed that the concentration of a 27 kDa TIMP protein corresponding to the molecular weight of TIMP-3 and migrating as previously reported between recombinant human TIMP-1 (30 kDa) and TIMP-2 (22 kDa) [36]; [37]; [38], decreased during G-CSF administration between days 0 and 6 and rebounded at day 8, 2 days after cessation of G-CSF administration (**Figure 1A–B**). 30 kDa TIMP-1 was slightly increased.

To determine whether TIMP-1 and -3 were transcribed in the BM, we quantified TIMP-1 and TIMP-3 mRNA from the central BM and the endosteal region by real-time RT-qPCR. TIMP-3 mRNA was expressed more abundantly at the endosteum than the central BM and G-CSF administration decreased TIMP-3 expression at both endosteum and central BM (**Figure 1C–D**). TIMP-1 mRNA expression was also more abundant at the endosteum than in the central BM (**Figure 1E–F**). However, unlike TIMP-3, TIMP-1 mRNA and protein were increased in the central BM and BM fluids during G-CSF administration (**Figures 1A and 1F**). Taken together, these data show that TIMP-3 expression in the BM is decreased at both mRNA and protein levels during G-CSF administration whereas TIMP-1 expression is slightly increased.

### High Expression of TIMP-3 in Stromal Cells at the Endosteum

**Figure 2:**
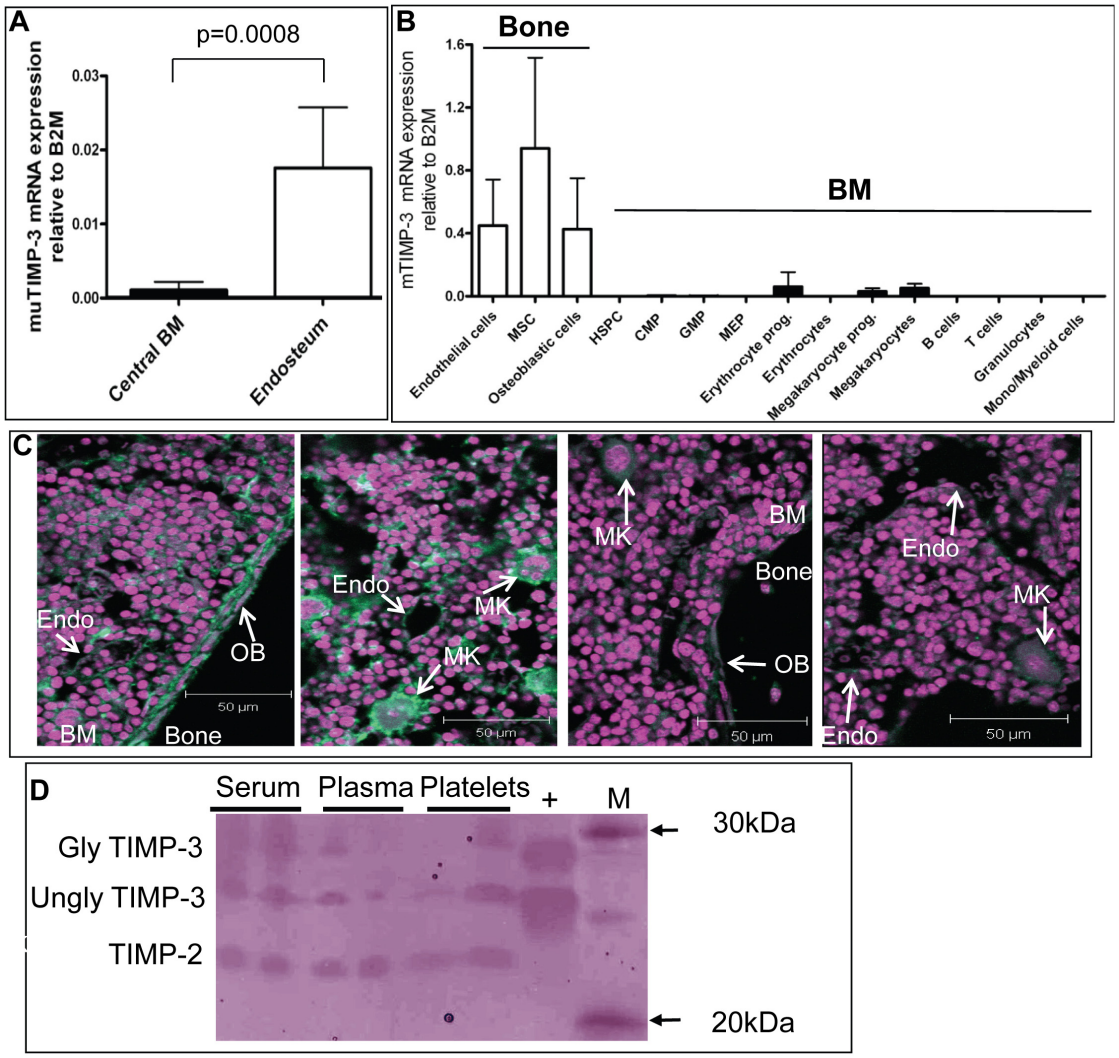
TIMP-3 expression pattern in the BM. (A) Comparison of TIMP-3 mRNA expression between endosteal and central BM cells by RT-qPCR. Data are mean ± SD of at least 7 mice per group normalized to B2M. (B) TIMP-3 mRNA in cells sorted from collagenase-treated compact bones or central bones. Phenotypes used are described in Supplementary Materials and Methods. Data are mean ± SD of at least 3 mice per group normalized to B2M. (C) TIMP-3 immunohistofluorescence by confocal laser scanning microscopy on bone sections x40 magnification. Two left panels are with rabbit anti-TIMP-3 antibody, two right panel with control non-immune rabbit IgG. Green color shows anti-TIMP-3 fluorescence whereas the pink color shows nuclei stained by DAPI. Osteoblasts (OB), endothelial cells (endo) and megakayocytes (MK) are indicated. (D) Reverse zymography of mouse blood plasma, sera and platelet lysates. Glycosylated and unglycosylated TIMP-3, and TIMP-2 are indicated. M lane contains molecular weight markers, + lane contains 20 ng of rmuTIMP-3.

We next explored TIMP-3 expression pattern by RT-qPCR in various cell populations from the BM. TIMP-3 was expressed at 10-fold higher levels in the endosteal region compared to central BM (**Figure 2A**). To further elucidate which endosteal cell populations most abundantly expressed TIMP-3, we sorted CD45^−^Lin^−^CD31^bright^ endothelial cells, CD45^−^Lin^−^CD31^−^Sca1^bright^ MSC with adipogenic, chondrogenic and osteogenic potential [39]; [40]; [41], and CD45^−^Lin^−^CD31^−^Sca1^−^CD51^+^ osteoblast lineage cells from endosteal cells extracted by collagenase treatment of bone fragments washed of the central BM [39]; [40]; [41]. Endothelial cells, MSC and osteoblastic cells all expressed high levels of TIMP-3 mRNA (**Figure 2B**).

In contrast, TIMP-3 expression in hematopoietic cells sorted from the central BM was very low to undetectable. Specifically Ter119^+^CD45^+^ erythroid progenitors, CD41^+^ megakaryocytes and CD41^+^KIT^+^ megakaryocyte progenitors expressed TIMP-3 at 10-fold lower levels compared to non-hematopoietic stromal cells. In HSPC such as Lin^−^Sca1^+^KIT^+^ (**LSK**) HSPC, common myeloid progenitors (**CMP**), granulocyte-monocyte progenitors (**GMP**) and megakaryocyte-erythrocyte progenitors (**MEP**), TIMP-3 expression was less than 1/100^th^ of stromal cells. TIMP-3 mRNA was undetectable (Ct>45) in the lineage positive leukocytes such as B220^+^ B cells, CD3^+^ T cells, Gr-1^bright^ granulocytes, and CD11b^+^Gr1^−^ myeloid cells (**Figure 2B**).

Immunohistofluorescence on fixed bone sections confirmed TIMP-3 expression in bone lining osteoblasts, endothelial cells as well as in megakaryocytes (**Figure 2C**).

Expression of TIMP-3 in megakaryocytes led us to examine TIMP-3 protein in blood plasma and platelets. Reverse zymography revealed that mouse blood plasma, serum and platelet lysates contained functional TIMP capable of inhibiting MMP-2 degradation of gelatin and migrating at same apparent molecular weight as commercial purified rmuTIMP-3 containing both 27 kDa glycosylated TIMP-3 and 24 kDa unglycosylated TIMP-3 [36] (**Figure 2D**). Therefore, while TIMP-3 in the BM could be essentially produced by BM stromal cells, particularly MSC, osteoblasts and endothelial cells, blood TIMP-3 is more likely to be released from mature platelets.

### Generation of Retroviral Vector to Overexpress huTIMP-3 in Murine Hematopoietic Cells *in Vivo*

**Figure 3:**
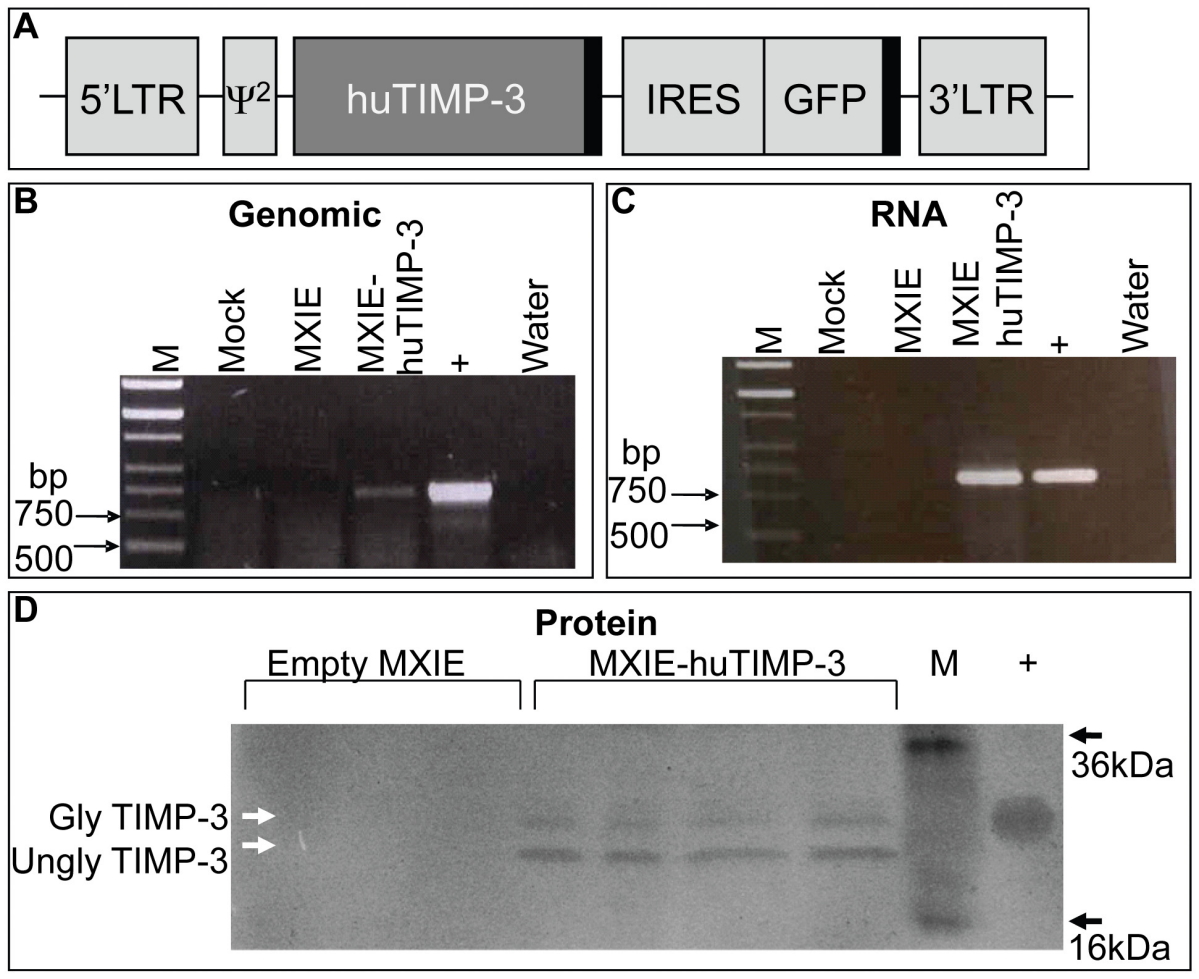
Retroviral vector to over-express huTIMP-3 in murine hematopoietic cells. (A) Representation of the MXIE retroviral vector containing huTIMP-3, an IRES, and GFP thus allowing for the co-expression of huTIMP-3 and GFP. (B) PCR and (C) RT-PCR of genomic DNA and RNA respectively from retrovirally transduced mouse hematopoietic cell line. M is markers, Mock are non-transduced cells, MXIE are cells transduced with empty-MXIE vector and MXIE-huTIMP-3 are cells transduced with the MXIE-huTIMP3 vector. + lane is the original plasmid containing huTIMP-3 cDNA as positive control. (D) Reverse zymography of culture supernatants from different clones of FDCP1 cells transduced with empty MXIE or MXIE-huTIMP-3 vectors. M is molecular weight markers, and + is 25 ng rhuTIMP-3.

To study the role of TIMP-3 in hematopoiesis, full length huTIMP-3 cDNA was cloned into retroviral vector MND-X-IRES-eGFP (**MXIE**) a vector less susceptible to gene silencing compared to the earlier generation MSCV vector [42]. The intra-ribosomal entry site (IRES) enables translation of both huTIMP-3 and GFP from the same transcript. Thus, cells that expressed GFP also expressed huTIMP-3 (**Figure 3A**).

We first assessed the effectiveness of the MXIE vector by retrovirally transducing the mouse myeloid cell line FDC-P1. Following sorting of GFP^+^ transduced FDC-P1 cells, we could demonstrate by PCR with primers specific for huTIMP-3 genomic integration and RNA expression of huTIMP-3 in cells transduced with MIXIE-huTIMP-3 but not in cells transduced with empty MIXIE (**Figure 3B-C**). Reverse zymography showed that functional huTIMP-3 was secreted by FDC-P1 cells transduced with MXIE-huTIMP-3 but not by cells transduced with MXIE control vector (**Figure 3D**).

### huTIMP-3 Overexpression Increases Myelopoiesis and Decreases Lymphopoiesis *in Vivo*

We then used this vector to retrovirally transduce BM cells from 5-FU-primed B6.SJL-PtprcaPep3b/BoyJ mice and then transplanted these transduced cells into lethally irradiated C57BL/6 congenic recipients. Eight weeks after transplantation, 10–35% blood leukocytes were GFP^+^ (average 22%) in mice reconstituted with MXIE-transduced HSC, and 6-24% blood leukocytes were GFP^+^ (average 16%) in mice reconstituted with MXIE-huTIMP-3-transduced HSC. Fourteen weeks post-transplant, using huTIMP-3 specific primers we confirmed by RT-qPCR that mice transplanted with MXIE-huTIMP-3-transduced BM cells expressed huTIMP-3 in central BM and endosteal regions whereas it was undetectable in mice transduced with MXIE vector (**Figure S1A**). Forced huTIMP-3 expression did not alter endogenous muTIMP-3 expression (**Figure S1B**).

Fourteen weeks post-transplant, mice reconstituted with huTIMP-3-transduced HSPC did not show any significant difference in body weight, blood cell number, BM cell number from a flushed femur, spleen weight or splenocyte number compared to MXIE controls (**Figure S1C-G**).

**Figure 4:**
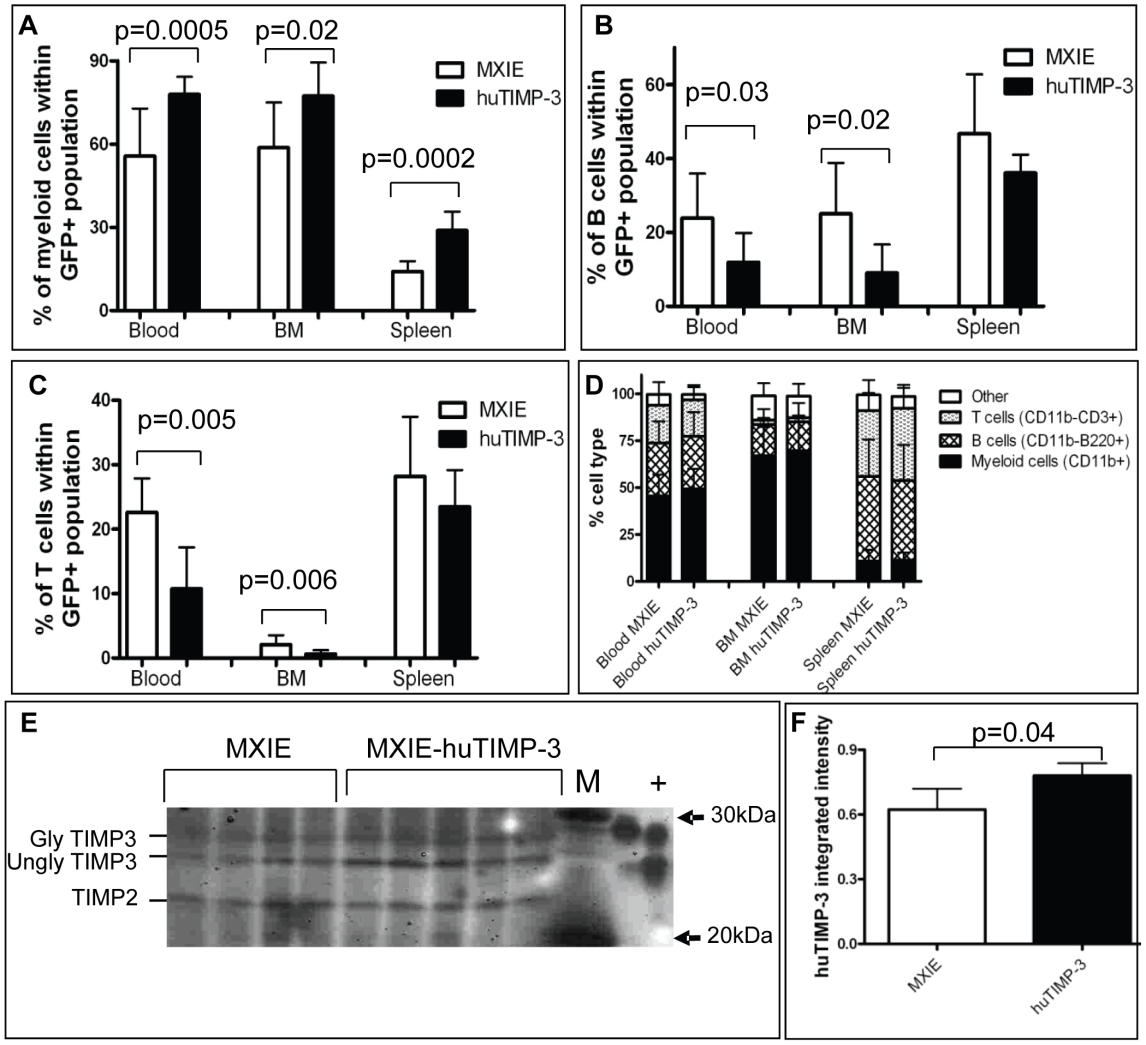
Flow cytometry analysis of leukocytes in mice over-expressing huTIMP-3 in hematopoietic cells . Frequency of CD11b^+^ myeloid cells (A), B220^+^ B cells (B) and CD3^+^ T cells (C) among GFP^+^ transduced cells in the BM, blood and spleen of mice reconstituted with HSC transduced with empty MXIE vector (white columns) or MXIE-huTIMP3 vector (black column). Data are mean ± SD of 9 mice per group. (D) Proportion of CD11b^+^ myeloid cells, B220^+^ B cells and CD3^+^ T cells in the BM, spleen and blood of the same mice when GFP expression was not taken into consideration. Data are mean ± SD of 9 mice per group. (E) Reverse zymography of BM fluids from mice transplanted with HSC transduced with empty MXIE vector or MXIE-huTIMP3 (n = 5 per group). M is molecular weight marker, + is 25 ng of rhuTIMP-3. (F) Quantification of the integrated intensity of TIMP-3 bands from panel E.

Lineage analysis by flow cytometry showed that in mice over-expressing huTIMP-3, CD11b^+^ myeloid cells were significantly over-represented amongst transduced GFP^+^ leukocytes in the BM, blood and spleen (**Figure 4A**). Conversely, the proportion of CD11b^−^B220^+^ B cells amongst transduced GFP^+^ huTIMP-3 over-expressing cells was significantly reduced in the blood and BM when compared to MXIE controls (**Figure 4B**). The proportion of CD3^+^ T cells among transduced GFP^+^ huTIMP-3 over-expressing cells was also significantly reduced in the blood and BM compared to MXIE controls (**Figure 4C**). Therefore, huTIMP-3 over-expression in hematopoietic cells favored myelopoiesis at the detriment of lymphopoiesis.

Unexpectedly, the frequencies of total (GFP^+^ and GFP^−^) myeloid, B and T cells were similar between the empty MXIE and MXIE-huTIMP-3 groups (**Figure 4D**). As TIMP-3 is not very soluble and is strongly associated with the ECM, entrapment of huTIMP-3 into the ECM surrounding huTIMP-3 secreting cells may have prevented huTIMP-3 diffusion to neighboring GFP^−^ cells which did not express huTIMP-3. To test the level of TIMP-3 secretion, reverse zymography was performed on 10 µg of total protein from BM fluid of retrovirally transduced mice. There was a significant but moderate 20% increase in the concentration of total free soluble functional TIMP-3 (of both mouse and human origins) compared to BM fluids from mice transduced with empty-MXIE (**Figure 4E–F**). Therefore, this 20% increase in total soluble TIMP-3 concentration in BM fluids, due to the retrovirally induced expression of huTIMP-3 over endogenous expression of muTIMP-3, may not have been sufficient to affect non-transduced hematopoietic cells.

### huTIMP-3 Overexpression Increases Colony Forming Cells (CFC) Trafficking

**Figure 5:**
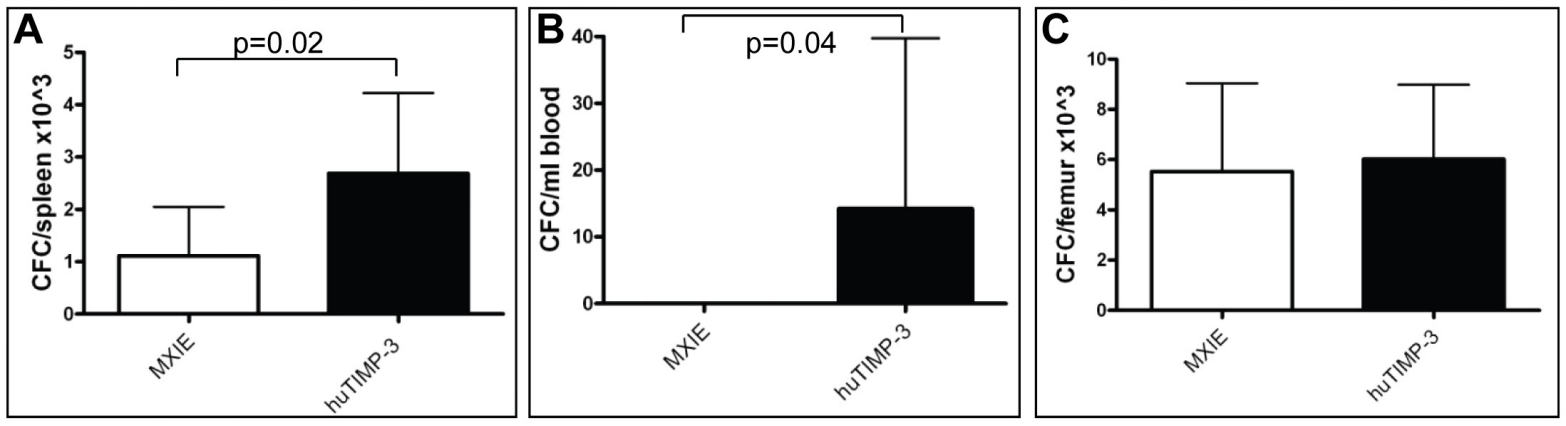
Increased CFC trafficking in mice over-expressing huTIMP-3. Colony-forming cell number in spleen (A), blood (B) and femoral BM (C) from mice transplanted with HSC transduced with empty MXIE vector (white columns) or MXIE-huTIMP3 vector (black columns). Data are mean ± SD of 9 mice per group.

As α1-antitrypsin related serpins affect HSPC trafficking [23]; [25], we tested if huTIMP-3 over-expression altered HSPC distribution between BM, blood and spleen. Colony assays showed that huTIMP-3 over-expression had no effect on the number of CFC per femur (**Figure 5C**). However, spleen from MXIE-huTIMP-3 mice contained 2.4-fold more CFC than spleens from mice transduced with empty MXIE (**Figure 5A**). Similarly CFC were detected in the blood of 4 out of 9 MXIE-TIMP3 mice while CFC were undetectable in the blood of 9 out of 9 control MIXE mice (**Figure 5B**, p = 0.04 Fisher's Exact test). This suggests that huTIMP-3 over-expression in hematopoietic cells slightly increases HSPC trafficking leading to accumulation in the spleen.

### huTIMP-3 Increases HSPC Proliferation *in Vivo* and *in Vitro*

**Figure 6:**
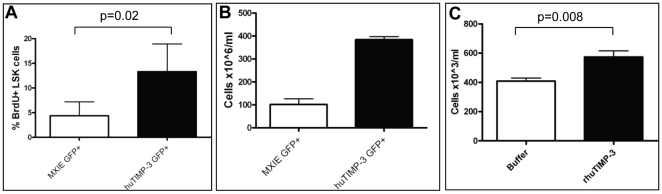
huTIMP-3 increases LSK cell proliferation *in vivo* and *in vitro*. (A) Proportion of transduced LSK cells that incorporated BrdU *in vivo* after 3 day BrdU administration. Data are mean ± SD of 5 mice per group. (B) *In vitro* proliferation of transduced LSK cells after 12 days of culture. LSK cells were seeded at 4,000/mL. Data are average ± SD of triplicates. (C) Day 7 effect of the addition of 200 ng/mL purified rhuTIMP-3 to liquid culture of LSK cells from non-manipulated mice. Data are mean ± SEM of 2 mice in quadruplicates.

In order to estimate HSPC turnover in mice over-expressing huTIMP-3, transplanted mice were given 5-bromo-2-deoxyuridine (**BrdU**) for three days before sacrifice. GFP^+^ LSK cells expressing huTIMP-3 had significantly increased BrdU incorporation *in vivo* (**Figure 6A**), demonstrating increased HSPC proliferation *in vivo*. This was confirmed when transduced GFP^+^ LSK cells were sorted from the BM of transplanted mice and cultured in serum-free medium in the presence of recombinant cytokines (ratKIT ligand, huIL-6, huIL-11 and huFlt3 ligand). After 12 days of cultures, huTIMP-3 over-expressing LSK expanded 2.7-fold more than LSK transduced with the control vector (**Figure 6B**).

To test whether this increased HSPC proliferation in mice over-expressing huTIMP-3 was a direct effect of TIMP-3, LSK cells were sorted from naïve C57BL/6 mice and cultured *in vitro* in absence or presence of 200 ng/mL rhuTIMP-3. After 7 days of culture, rhuTIMP-3 significantly increased LSK proliferation by 1.4 fold (**Figure 6C**). Thus, huTIMP-3 increases the proliferation of LSK cells both *in vivo* and *in vitro*.

### Fatal Osteosclerosis in Mice Overexpressing huTIMP-3

**Figure 7:**
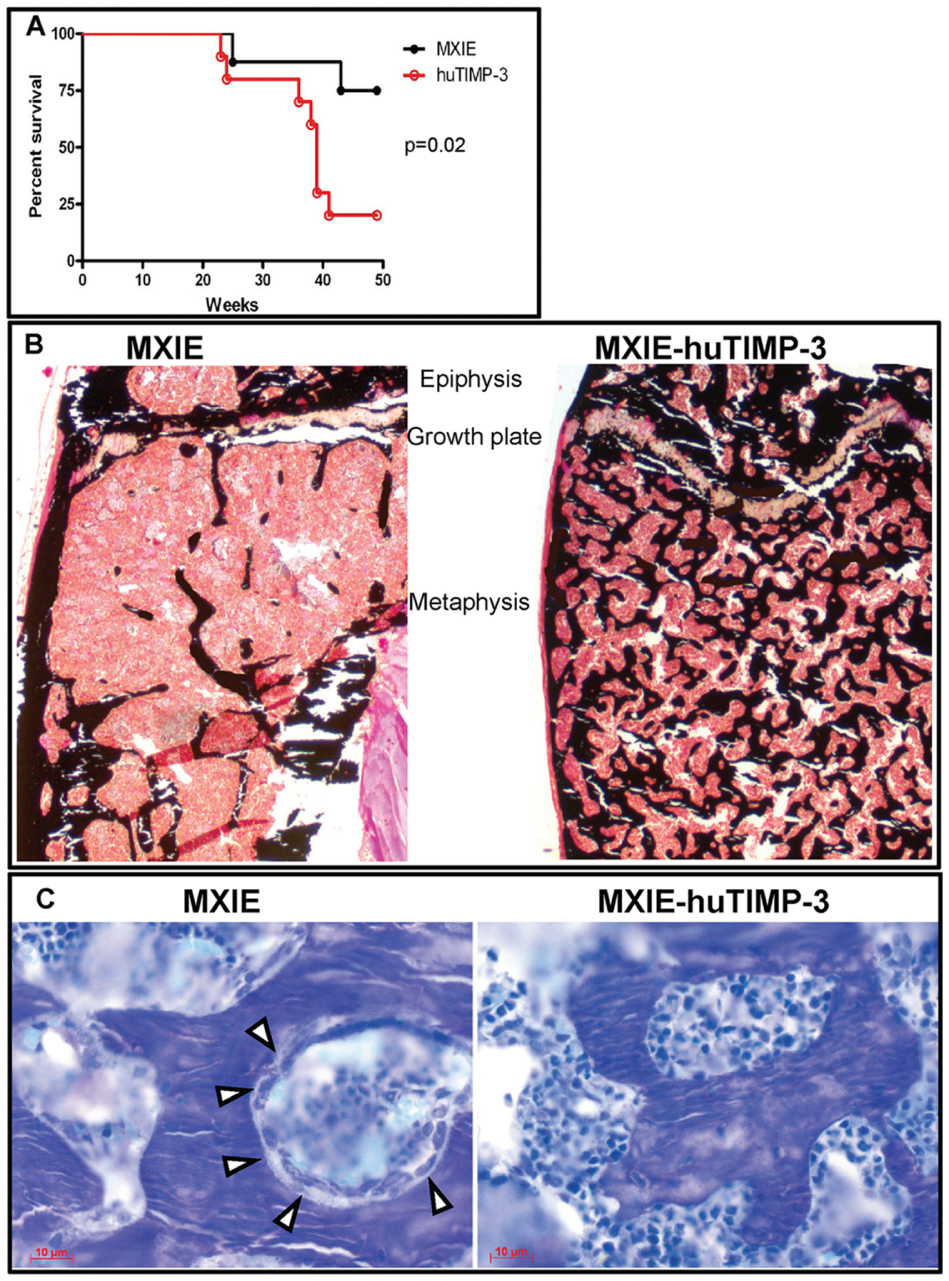
Long-term over-expression of huTIMP-3 in hematopoietic cells leads to fatal osteosclerosis. (A) Survival curve of mice transplanted with HSC transduced with empty MXIE vector or MXIE-huTIMP3 vector (n = 10 in each group). (B) von Kossa staining and toluidine blue staining (C) of tibial sections from mice transplanted with HSC transduced with empty MXIE vector or MXIE-huTIMP3 vector. In panel B, black staining shows calcified bone. In panel C, cuboidal osteoblasts covering the osteoid are indicated by empty arrow heads. Note that osteoblasts and osteoid are absent in mice transduced with MXIE-huTIMP3.

To determine the long-term effects of over-expressing huTIMP-3 in hematopoietic cells, a cohort of mice were maintained for a year. Unexpectedly, a significant proportion of mice transplanted with MXIE-huTIMP-3 HSPC died between 36 and 39 weeks after transplantation (**Figure 7A**). In MXIE-huTIMP-3 mice that died first, it was impossible to flush the BM as the BM cavity was filled with solid bone. Examination of the tibias and femurs from mice overexpressing huTIMP-3 that survived, revealed that the BM cavity was filled with calcified bone resulting in markedly decreased BM content (**Figure 7B–C**) with a large amount of trabecular bone in the metaphysis and epiphysis. In the regions where trabecular bone areas were increased, cortical bone was also highly porous, suggesting destruction or lack of development of the normal cortical structure. The excess bone was woven in nature and the endosteal surfaces were largely inactive; without any morphologically identifiable osteoblasts or osteoclasts on the bone surfaces (**Figure 7C**). No cartilage remnants were detected within the trabecular bone, indicating that this excess bone did not arise because of a lack of resorption of newly formed trabecular bone. Rather, it seems that an excessive amount of bone formation occurred at some time prior to sample collection but was not continuing at the time of death as osteoblasts were absent. Clearly the normal signaling pathways which would activate remodeling of the bone did not respond by increasing bone turnover to remove the excess bone, leading to a premature exhaustion of the osteoblastic lineage.

## Discussion

In embryos and early post-natal mice, TIMP-3 is expressed at sites of extensive tissue remodeling such as developing bony structures and somites, lung, skin, hair follicles, ear, external regions of the eye, cornea, choroid plexus as well as interdigit webs [43]. In adult mice, TIMP-3 mRNA and protein were detected in a the kidney cortex, liver, spleen, muscle, heart, brain, ovarian follicles, testis and hair follicles [43]. Relevant to our study, TIMP-3 was also found to be expressed in adult bones but its expression in the hematopoietic system has not been investigated [43].

We report that TIMP-3 is highly expressed in the endosteal region compared to central BM. Endothelial cells, MSC and osteoblastic cells, which are all important cellular components of HSC niches [6]; [7]; [44]; [45]; [46]; [47], express high levels of TIMP-3. Megakaryocytes, their progenitors, and erythroid progenitors also expressed TIMP-3 mRNA at much lower levels. At the protein level, megakaryocytes and platelets were positive for TIMP-3. This is consistent with previous reports showing that fibroblasts, erythroid and megakayrocyte progenitors derived from human long-term marrow cultures express TIMP-3 [48] and that huTIMP-1, -2 and -3 are present in platelets [49]. Myeloid cells, lymphoid cells, Lin^−^ myeloid progenitors and LSK HSPC tested showed no detectable TIMP-3 expression. As non-hematopoietic stromal cells at the endosteum express high levels of this protein, TIMP-3 could be an important component of HSPC niches and regulate hematopoiesis *in vivo*. This is supported by 1) our observation that TIMP-3 expression is reduced during G-CSF-induced mobilization and 2) that TIMP-3 is expressed in hair follicle bulges that form specific niches for epidermal stem cells and its expression is up-regulated when quiescent epidermal stem cells shift towards a proliferative state [50]. Furthermore, we show herein that over-expression of huTIMP-3 in hematopoietic cells *in vivo* 1) increases LSK cell proliferation, 2) favors myelopoiesis at the detriment of lymphopoiesis 3) increases trafficking of CFC to the spleen and 4) results in late onset fatal osteosclerosis whereby most of the BM cavity was filled with mineralized bone, demonstrating that TIMP-3 is a key regulator of both hematopoiesis and bone turn-over.

Our data showing increased BrdU incorporation in LSK cells following in vivo or in vitro exposure to TIMP-3 are consistent with a recent report showing that in vivo overexpression of TIMP-3 in mice for a period of 8 days by hydrodynamic transfection caused increased HSC proliferation with increased production of multipotent progenitors independently of TIMP-3 protease inhibitory domain [35]. As a result, hydrodynamic transfection of TIMP-3 in 5-FU treated mice accelerated recovery whereas TIMP-3 deficient mice had blunted recovery from 5-FU [35]. However these authors did not investigate longer term effects of TIMP-3 overexpression on myelopoiesis, HSPC trafficking and bone formation.

TIMP-1 and TIMP-2 are known to have erythroid-potentiating activity [26] and promote proliferation of a variety of human and mouse hematopoietic and non-hematopoietic cell lines *in vitro* [27]; [28]. Therefore, growth promoting activity of TIMP-3 *in vitro* may be a similar feature of all TIMP proteins. However unlike TIMP-3, over-expression of TIMP-1 or TIMP-2 in hematopoietic cells using a MSCV retroviral vector similar to MXIE did not affect murine hematopoiesis *in vivo* [29]. Although huTIMP-3 over-expression using the MXIE retroviral vector was not sufficiently high to cause global effects on BM hematopoiesis, our data show that LSK HSPC overexpressing huTIMP-3 proliferate faster and traffic more with their differentiation skewed towards myelopoiesis at the detriment of lymphopoiesis *in vivo*. Because TIMP-3 has multiple functions in addition to MMP inhibition, the unique effects of TIMP-3 over-expression on hematopoiesis *in vivo* compared to the lack of effect of TIMP-1 or TIMP-2 over-expression is likely to lie beyond TIMP-3 MMP inhibitory activity, in unique functions that TIMP-1 and TIMP-2 do not possess.

Due to unique basic domains both at the C- and N-terminus of TIMP-3, the majority of the protein is bound to the ECM, whereas the other members of the TIMP family are found in a soluble form [30]; [31]; [32]. In addition to inhibiting soluble MMP, TIMP-3 is also able to inhibit membrane bound and transmembrane MMP [51]; [52]. The long-term over-expression of huTIMP-3 in murine hematopoietic cells resulted in late onset and dramatic osteosclerosis, eventually resulting in death. The volume of trabecular bone depends on the coordinated actions of osteoblasts (bone forming cells) and osteoclasts (bone destroying cells). The increased bone volume must be caused either by inhibited osteoclastic bone resorption or increased bone formation, but the exact cause is unclear. The lack of cartilage remnants and the woven bone present in the aged TIMP-3 over-expressing samples suggests that there has been an excess of bone formation. This was surprising since most functions of MMPs, especially MMP-2, MMP-9 and MT1-MMP, have focused on their essential roles as bone-degrading proteases required for osteoclast function [53]; [54]; [55]. Furthermore, mice lacking MMP-13 are reported to have increased bone volume due to decreased osteoclast formation and function, but also demonstrate enhanced osteoblast function [56]; [57]. TIMP-1 and TIMP-2 inhibit bone resorption *in vitro*; and in young mice, TIMP-1 over-expression reduces bone resorption resulting in increased bone mass [58]; [59]. Synthetic inhibitors of MMPs and TIMP-2 can also inhibit osteoclast migration to bone resorption site, thus decreasing bone remodeling [60].

The lack of osteoblast in mice over-expressing huTIMP-3 in hematopoietic cells may have occurred due to over-stimulation and proliferation of osteoblast progenitors following transplant, leading to a massive burst of bone formation followed by the exhaustion of the osteoblastic lineage, resulting in their complete loss from endosteal surfaces. Therefore, although osteoblasts and their MSC ancestors express TIMP-3, excessive TIMP-3 may cause excessive proliferation and differentiation towards the osteoblastic lineage and exhaustion of the mesenchymal/osteoblastic cell reserve in the BM. Because osteoblast lineage cells and MSC are an integral part of the HSC niche, their disappearance may have compromised HSC niches within the BM, leading to decreased hematopoiesis and ultimately death. The progressive and eventually fatal osteosclerosis may be responsible for the slight but significant increase in splenic hematopoiesis 14 weeks following transplantation of HSPC transduced with huTIMP-3. This late onset osteoclerosis could be due to the relatively small enhancement of huTIMP-3 expression over endogenous muTIMP-3 expression.

Another unique property of TIMP-3 is its ability to inhibit angiogenesis by specifically blocking the binding of VEGF-A to its receptor VEGF-R2. This inhibition prevents the phosphorylation of VEGF-R2 and down-stream signaling events that would normally promote vascular formation [34]. TIMP-3 can reduce circulating endothelial progenitors and decrease neovascularization in tumors both *in vitro* and *in vivo* [61]; [62]; [63]. VEGF-A has also been found to be a chemo-attractant for osteoclasts, essential for osteoclast migration and invasion and promote osteoclast bone resorption [54]; [64]. Therefore, the ability of huTIMP-3 to affect VEGF-A activity and binding may also play a role in the osteosclerosis seen in mice transplanted with huTIMP-3 transduced BM cells.

Interestingly, Nakajima et al have recently reported that similar to VEGFR2, TIMP-3 can directly bind to the receptor tyrosine kinase Tie2 expressed by HSC, inhibiting its phosphorylation in response to its ligand angiopoietin-1 resulting in HSC release from quiescence [35]. As angiopoietin-1 is expressed by osteoblast-lineage cells, MSC and to a lower extent by endothelial cells at the endosteum [41], it is also possible that part of the effect we observed is mediated by inhibition of Tie2-mediated signaling.

Finally, TIMP-3 has the unique property amongst TIMP family to inhibit membrane bound sheddases of the ADAM family, particularly TACE (or ADAM-17), which converts membrane-bound pro-TNFα into active soluble TNFα [33]. By inhibiting TACE activity and inhibiting TNFα maturation, TIMP-3 is an important negative regulator of TNFα. Deletion of the TIMP-3 gene in mice leads to chronic hepatic inflammation, systemic inflammation after lipospolysaccharide administration and death due to uncontrolled TNFα activation [33]; [65]; [66]. Importantly Cre-mediated conditional inactivation of TACE gene in osteochondroprogenitors via the *Sox9* promoter leads to decreased B cells and T cells, increased granulocytes, extramedullary hematopoiesis in the spleen and liver as well as increased LSK cell number in the BM [67]. The phenotype in this mouse model is very similar to what we observed by over-expressing huTIMP-3 in haematopoietic cells *in vivo*. Additionally, TACE processes a number of trans-membrane proteins controlling HSPC functions such as IL-6 receptor [68], Notch-1 [69], Notch ligand Delta 1 [70], CSF-1 receptor [71], VCAM-1 [72], c-KIT [73] and Flt3-L [74]. Therefore, the effect of huTIMP-3 over-expression may not be limited to the alteration of TNF-α activation in the BM but also include decreased processing of these important hematopoietic cytokines expressed in BM niches. Therefore, it is tempting to speculate that part of the effect of huTIMP-3 overexpression on HSPC proliferation, myelopoiesis, lymphopoiesis and trafficking to the spleen could be due to TIMP-3-mediated inhibition of TACE in osteochondroprogenitors forming HSC niches.

In conclusion, our results suggest that TIMP-3 is an important component of HSC niches and regulates both HSC and bone turn-over. This is supported by our finding that 1) most TIMP-3 is expressed at the endosteum by stromal cells known to form HSC niches, 2) TIMP-3 mRNA and protein expression decreases under hematopoietic system stress such as G-CSF induced mobilization, 3) over-expression of huTIMP-3 increases HSPC proliferation in the BM, 4) favors myelopoiesis at the detriment of lymphopoiesis and 5) increases bone formation while decreasing bone resorption *in vivo*.

## Materials and Methods

### Mice

All procedures were approved by the Animal Ethics Experimentation Committee of the University of Queensland, Australia.

Mice used for TIMP-3 localization studies were 12-14 wk male C57BL/6. For mobilization experiments, 8 week-old male C57BL/6 mice were subcutaneously injected twice daily with a total of 250 µg/kg/day of recombinant human G-CSF (Neupogen) diluted in saline.

In the transplant studies, 9 week old male B6.SJL-PtprcaPep3b/BoyJ BM (CD45.1^+^) were retrovirally transduced to over-express huTIMP-3 and transplanted into 11 wk female C57Bl/6 (CD45.2^+^). For late effect of TIMP-3 over-expression, HSC donors and recipients were 8.5 week 129Sv. All mice were purchased from the Animal Resource Centre (Western Australia, Australia).

### Tissue Harvesting

On the day of sacrifice, mice were weighed and anesthetized with isofluorane for cardiac puncture prior to sacrifice by cervical dislocation. Blood was collected into 20 U of heparin before red cell lysis in 0.15 M NH_4_Cl, 10 mM NaHCO_3_, 1 mM EDTA pH = 7.4 buffer. Hips, single femur and spine were surgically removed, cleaned and placed into a tube containing PBS supplemented with 2% newborn calf serum (NCS) for crushing. The remaining femur was flushed with 1 mL PBS and the emptied bone was kept for further RNA extraction. BM fluid was collected after pelleting the femur flush at 370 g and stored at -70°C. Spleens were weighed before being dissociated in PBS with 2% NCS. Tibias were fixed in ice cold PBS containing 4% paraformaldehyde and rotated overnight at 4°C. A single tibia was allowed to decalcify in 10% EDTA for 2-3 weeks before paraffin embedding.

### Quantitative Real-Time RT-PCR (RT-qPCR)

2–4×10^6^ BM cells from the flushed femur were extracted with 1 mL Trizol (Invitrogen, USA). Endosteal RNA was collected by flushing the empty femur with 1 mL Trizol to extract cells adjacent to the bone. RNA was reverse transcribed using random hexamers (Roche, Switzerland) and SuperScriptIII (Invitrogen, USA). Primers were designed to cross intron-exon boundaries that do not amplify genomic DNA. Taqman probe “mu/huTIMP3 probe 639b” was used in conjunction with mouse (mu) specifc or human (hu) specific TIMP-3 primers (**Table 1**). The probe was labeled with 5′ FAM and 3′ BHQ1. RT-qPCR reactions were performed following manufacturer's instructions using 8 µL of a 1/20 dilution of cDNA (ABI systems/Invitrogen, USA). Samples were standardized to the house-keeping gene β2-microglobulin (B2M) and amplified with specific primers (**Table 1**).

**Table 1:** RT-qPCR primers used for mouse and human TIMP-3 analysis.

muTIMP3 564f	5′-ACACGGAAGCCTCTGAAAGTC-3′
muTIMP3 691b	5′-ACTTTGTGGAGAGGTGGGAC-3′
huTIMP-3 255f	5′-AGCTTCCGAGAGTCTCTGTG -3′
huTIMP-3 371b	5′-CACCTCTCCACGAAGTTGC-3′
mu/huTIMP3 probe 639b	5′-ACGCGCCCTGTCAGCAGGTA-3′
muB2M 75f	5′-CTGGTCTTTCTGGTGCTTGTC -3′
muB2M 181b	5′-GTATGTTCGGCTTCCCATTC-3′
muB2M probe 94f	5′-CACTGACCGGCCTGTATGCTATCCA-3′

### Colony Assays

Leukocytes from BM, blood and spleens were counted on a Sysmex KX-21 automated cell counter. Colony assays, done in duplicates, were performed with 2×10^5^ cells plated in 35 mm Petri dishes containing 1 mL of Iscove's Modified Dulbecco's Medium (**IMDM**) with 1.62% methylcellulose (Fluka, USA), 30% fetal calf serum and saturating doses of recombinant muIL-3, muIL-6 and muSCF conditioned media from stably transfected X63 and BHK cell lines. Colonies were counted after 12 days of culture at 37°C with 5% CO_2_.

### Flow Cytometry Staining and Cell Sorting

To sort CMP, GMP and MEP from the BM, the following stain was performed in PBS with 2% NCS: Biotinylated anti-lineage (Lin) antibody cocktail (CD3, CD5, B220, Gr1, F4/80, Ter119) and biotinylated anti-IL7Rα with streptavidin (**SAV**)-Pacific Blue, CD34-fluorescein isothiocyanate (**FITC**), CD16/32-phycoerythrin (**PE**), Sca-1-phycoerythrin-cyanine 7 (**PECY7**) and Kit-allophycocyanin (**APC**). The populations were defined as follow: Lin^−^IL7Rα^−^Kit^+^Sca-1^−^CD34^+^CD16/32^−^ (CMP), Lin^−^IL7Rα^−^Kit^+^Sca-1^−^CD34^+^CD16/32^+^ (GMP) and Lin^−^IL7Rα^−^Kit^+^Sca-1^−^CD34^−^CD16/32^−^ (MEP) [75].

All other stains were performed in CD16/CD32 hybridoma 2.4G2 supernatant (Fc block) to block IgG Fc receptors. BM cells stained with CD11b-PE and Gr-1-FITC were sorted for monocyte/myeloid cells (CD11b^+^Gr-1^−^) and granulocytes (CD11b^+^Gr-1^bright^). Megakaryocytes (CD45^+^CD41^+^), pro-erythroblasts (CD45^+^Ter119^+^) and their KIT^+^ progenitors were stained and sorted using Ter119-FITC, Kit-PE, CD45-allophycocyanin-cyanine 7 (**APCCY7**) and CD41-biotin with SAV-Pacific Blue. BM cells stain with B220-FITC, CD11b-PECY7 and CD3-Peridinin chlorophyll protein 5.5 (**PerCPCY5.5**) were sorted for B cells (CD11b^−^B220^+^) and T cells (CD11b^−^CD3^+^). Populations collected were mixed in Trizol for qRT-PCR analysis.

To sort endosteal cells associated with the bone, cleaned hips, femurs, tibias and spine were crushed and BM cells were discarded. Following treatment with 3 mg/mL collagenase type I from C. histolyticum (Worthington, USA) for 40 min at 37°C, bone cells were washed through a 40 µm filter, pelleted at 370 g and resuspended in Fc block. Biotinylated lineage antibodies (CD3, CD5, Gr-1, B220, CD11b and Ter119) and SAV-conjugated magnetic activated cell sorting (MACS) beads were used to magnetically deplete mature hematopoietic cells. Lineage negative cells were then stained with SAV-FITC, CD51-PE, Sca-1-PECY7, CD31-APC and CD45-APCCY7 and sorted. The following populations were collected: endothelial cells (CD45^−^Lin^−^CD31^bright^), MSC (CD45^−^Lin^−^CD31^−^CD51^+^Sca-1^bright^) and osteoblast/osteoprogenitors (CD45^−^Lin^−^CD31^−^CD51^+^ Sca-1^−^) [76]; [77]; [78]. Cells were spun down and resuspended in Trizol containing 0.1 µg/mL tRNA.

To isolate LSK HSPC, BM cells were stained with biotinylated lineage antibodies (CD3, CD5, B220, CD11b, Gr-1, CD41, Ter119) and SAV-APCCY7, Sca1-PE and Kit-APC. LSK cells collected for *in vitro* culture were grown in X-Vivo supplemented with 0.1% human serum albumin (CSL, Australia), rhuIL-6 (10 ng/mL), rhuIL-11 (10 ng/mL), rratKIT ligand (100 ng/mL) and rhuFlt3 ligand (50 ng/mL). Recombinant huTIMP-3 (R&D, USA) was added at a concentration of 200 ng/mL and the storage buffer was used as control (25 mM Tris, 0.15 M NaCl pH 7.5). Mice used for BrdU incorporation were given an intra-peritoneal injection of 100 mg/kg BrdU and their water was supplemented with 1 g/L BrdU three days prior to sacrifice. Sorted LSK cells were cytospun onto glass slides, acetone fixed and BrdU stained as per manufacturer's protocol (BD Pharmingen, USA). BrdU slides were counted blind.

For analysis of the donor/host chimerism, BM, spleen or blood cells were pelleted at 370 g for 5 minutes at 4°C and resuspended in Fc block. B cell chimerism was analyzed with CD45.1-PE, CD11b-PECY7, B220-APCCY7 and CD19-Biotin-SAV-PerCPCY5.5; myeloid and T cell chimerism with CD45.1-PE, CD11b-PECY7, CD45.2-PerCPCY5.5, Gr-1-APCCY7 and CD3-biotin with SAV-APC. LSK cells were stained with biotinylated lineage antibodies (CD3, CD5, B220, CD11b, Gr-1, CD41, Ter119) and SAV-PerCpCY5.5, CD150-PE, Sca1-PECY7, Kit-APC and CD48-Pacific Blue.

Cell sorting was performed on the BD FACSAria Cell-Sorting System (USA). FACS analysis data was collected on BD's LSRII System and analyzed using FlowJo (Tree Star, USA). All antibodies were from BD Pharmingen except CD48-Pacific Blue, CD41-Biotin and CD150-PE from BioLegend.

### Isolation of Serum, Plasma and Platelets

For serum collection, whole blood was left at room temperature for 1 hr and then ice for 30 min. Blood for plasma and platelet lysis was collected in citrate dextrose and spun at 735 g for 20 min. The supernatant was re-spun at 1,310 g for 30 min. The supernatant (plasma) was removed and pelleted platelets were lysed on ice for 35 min in 50 mM Tris pH 7.5 and 1%NP40 contain the inhibitors pepstatin A (1 µg/mL), leupeptin (2 µg/mL), E64 (5 µg/mL), aprotinin (10,000 U/mL) and phenylmethylsulfonyl fluoride (0.2 mM) before centrifugation at 15,000 g. All supernatant were frozen at -70°C.

### Reverse Zymography

Samples of equal protein concentration, as determined by Micro BCA Protein Assay Kit (Pierce), were run in a 15% polyacrylamide gel containing 2.25 mg gelatin (Ajax, Australia) and 10 µg rhuPro-MMP-2 (R&D). The gel was washed in 2.5% TritonX100 and incubated overnight at 37°C in a buffer containing 50 mM Tris pH 7.5, 200 mM NaCl, 5 mM CaCl_2_ and 0.02% Brij-35. Activated MMP-2 degraded its substrate gelatin except for areas where TIMPs were present. Areas of MMP inhibition were visible following Coomassie Blue staining. Positive control was either 25 ng of rhuTIMP-3 (R&D) or 20 ng of rmuTIMP-3 (Abcam). Integrated intensity of TIMP-3 bands were quantified using the 700 channel on Odyssey Infrared Imaging System (Li-Cor Biosciences).

### Immunohistofluorescence

Standard dewaxing and immunofluorescence protocol was performed before following manufacturer's instructions for Tyramide Signal Amplification (TSA) Kit (Perkin-Elmer). Polyclonal rabbit anti-muTIMP3 primary antibody (Abcam) or purified non-immune rabbit IgG control (Jackson ImmunoResearch Laboratories) were used at 10 µg/mL, followed by donkey F(ab)_2_ anti-rabbit IgG conjugated to horse radish peroxidase (Jackson ImmunoResearch Laboratories). Biotinyl Tyramide working solution was incubated for 8 minutes before washing and incubation with streptavidin-AlexaFluor488 (Invitrogen). Samples were mounted in ProLongGold containing 0.25 µg/mL DAPI and analyzed on Zeiss LSM 510 Meta confocal laser scanning microscope with the oil 40x objective. DAPI fluorescence was excited at 405 nm with a diode laser and detected through a 420–480-nm band-pass filter. AlexaFluor 488 was excited at 488 nm with an argon laser and emission was detected through a 505–530-nm band-pass filter. Images are average of 16 consecutive scans and were analyzed using LSM 510 software (Zeiss, Germany).

### Bone Histology

Undecalcified tibias and femurs were fixed overnight in 4% paraformaldehyde, transferred into 70% ethanol and embedded in methacrylate as previously described [79]. Undecalcified 5 µm sections were stained with toluidine blue or von Kossa [79].

### Vector Construction

The coding region of huTIMP-3 was amplified from a plasmid kindly provided by Dr MC Rio (IGBNC, France) using DeepVent (New England Biolabs, USA). The primers used were as follow: huTIMP-3-279f 5′-AGAATTCAGATCTGCAGCGGCAATGACCCCTTGG-3′ (containing additional EcoRI and BglII restriction sites upstream of the huTIMP-3 kozak sequence and start codon) and huTIMP-3-937b 5′-CCCTGAGCGCCAGACCCTGCCTCGAGTC-3′ (containing the huTIMP-3 stop codon followed by an XhoI restriction site). DNA sequence integrity was confirmed using Big Dye Terminator v3 (Applied Biosystems, USA), huTIMP-3 cDNA was cloned into dephosphorylated MXIE [42] at the EcoRI restriction site. Ligation was performed using T4 DNA Ligase (Promega, USA) as per manufacturer's instruction and plasmid was electroporated into JM109 bacterial cells. Insert orientation was determined by restriction digest with HindIII.

### Retroviral Transduction

Empty MXIE or MXIE-huTIMP-3 plasmids linearised with ScaI, and HindIII-lineared DsRed vector containing a neomycin resistance cassette, were co-transfected into the ecotropic packaging cell line GP+E-86 with Metafectene (Biontex, Germany). Cells were incubated in Dulbecco's Modified Eagle Medium supplemented with 10% FCS overnight. G418 (0.5 mg/mL) was added to select stable transfectants. After 2 weeks of selection, transfected GP+E-86 cells expressing GFP from MIXIE vectors were sorted by FACS and seeded in a clonal fashion in 96-well plates. The highest GFP expressing stable transfectant clones were irradiated at 15Gy to stop replication capacity and used to transfect mouse myeloid cell line FDC-P1, or mouse BM cells by a 3 day co-culture. RNA and genomic DNA were collected for PCR using the cloning primers listed above, cell lysate and supernatant were collected for reverse zymography.

### Transplantation of Retrovirally Transduced HSC

Donor B6.SJL-PtprcaPep3b/BoyJ mice were injected intraveinously 150 mg/kg 5-fluorouracil. Mononucleated BM cells were collected from four days later and co-cultured for 3 days with irradiated GP+E-86 packaging cells transfected with either empty MXIE or MXIE-huTIMP-3 plasmids in a ratio of five BM cells to one packaging cell in IMDM supplemented with 15% FCS, 4 µg/mL polybrene (Sigma Chemicals), 100 ng/mL rratSCF, 50 ng/mL rhuFlt3L, 10 ng/mL rhuIL-6 and 10 ng/mL rhuIL-11 (Preprotec). Post culture, 1×10^6^ transduced BM cells were washed and retro-orbitally injected into C57BL/6 mice lethally irradiated with two split doses of 5.5Gy each 24 hours prior to transplant. Mice were bled via the tail vein at 8weeks post-transplant to assess engraftment levels by flow cytometry (CD45.1 vs CD45.2 and GFP positivity).

### Statistics

Significance levels were calculated using the Mann-Whitney test in GraphPad Prism v5.01. Survival statistics was calculated in the same program using the Log-rank (Mantel-Cox) test.

## Supporting Information

Figure S1 Lack of effect of huTIMP-3 overexpression in hematopoietic cells on mouse weight and white blood cell counts in blood, BM and spleen 14 weeks post-transplant. RT-qPCR for huTIMP-3 expression (A) and endogenous muTIMP-3 expression (B) in central BM cells and endosteal cells from mice transplanted with HSC transduced with empty MXIE vector or MXIE-huTIMP3 vector. Data are normalized to B2M mRNA and are mean±SD of 5 mice per group. UND is for undetected. Body weight (C), spleen weight (D), white cell counts in spleen (E), femur (F) and blood (G). Data are mean±SD of 9 mice per group.
(0.77 MB TIF)

## References

[1] Nilsson SK, Johnston HM, Coverdale JA (2001). Spatial localization of transplanted hemopoietic stem cells: inferences for the localization of stem cell niches.. Blood.

[2] Kohler A, Schmithorst V, Filippi MD, Ryan MA, Daria D (2009). Altered cellular dynamics and endosteal location of aged early hematopoietic progenitor cells revealed by time-lapse intravital imaging in long bones.. Blood.

[3] Lo Celso C, Fleming HE, Wu JW, Zhao CX, Miake-Lye S (2009). Live-animal tracking of individual haematopoietic stem/progenitor cells in their niche.. Nature.

[4] Xie Y, Yin T, Wiegraebe W, He XC, Miller D (2009). Detection of functional haematopoietic stem cell niche using real-time imaging.. Nature.

[5] Nakamura Y, Arai F, Iwasaki H, Hosokawa K, Kobayashi I (2010). Isolation and characterization of endosteal niche cell populations that regulate hematopoietic stem cells.. Blood.

[6] Kiel MJ, Yilmaz OH, Iwashita T, Yilmaz OH, Terhorst C (2005). SLAM family receptors distinguish hematopoietic stem and progenitor cells and reveal endothelial niches for stem cells.. Cell.

[7] Sugiyama T, Kohara H, Noda M, Nagasawa T (2006). Maintenance of the hematopoietic stem cell pool by CXCL12-CXCR4 chemokine signaling in bone marrow stromal cell niches.. Immunity.

[8] Trumpp A, Essers M, Wilson A (2010). Awakening dormant haematopoietic stem cells.. Nat Rev Immunol.

[9] Wilson A, Trumpp A (2006). Bone-marrow haematopoietic-stem-cell niches.. Nat Rev Immunol.

[10] Kiel MJ, Morrison SJ (2008). Uncertainty in the niches that maintain haematopoietic stem cells.. Nat Rev Immunol.

[11] Askmyr M, Sims NA, Martin TJ, Purton LE (2009). What is the true nature of the osteoblastic hematopoietic stem cell niche?. Trends in endocrinology and metabolism: TEM.

[12] Lévesque JP, Helwani FM, Winkler IG (2010). The “osteoblastic” niche and its role in hematopoietic stem cell homing and mobilization.. Leukemia in press.

[13] Lévesque JP, Takamatsu Y, Nilsson SK, Haylock DN, Simmons PJ (2001). Vascular cell adhesion molecule-1 (CD106) is cleaved by neutrophil proteases in the bone marrow following hematopoietic progenitor cell mobilization by granulocyte colony-stimulating factor.. Blood.

[14] Lévesque JP, Hendy J, Takamatsu Y, Williams B, Winkler IG (2002). Mobilization by either cyclophosphamide or granulocyte colony-stimulating factor transforms the bone marrow into a highly proteolytic environment.. Exp Hematol.

[15] Petit I, Szyper-Kravitz M, Nagler A, Lahav M, Peled A (2002). G-CSF induces stem cell mobilization by decreasing bone marrow SDF-1 and up-regulating CXCR4.. Nat Immunol.

[16] Lévesque JP, Hendy J, Takamatsu Y, Simmons PJ, Bendall LJ (2003). Disruption of the CXCR4/CXCL12 chemotactic interaction during hematopoietic stem cell mobilization induced by GCSF or cyclophosphamide.. J Clin Invest.

[17] Lévesque JP, Hendy J, Winkler IG, Takamatsu Y, Simmons PJ (2003). Granulocyte colony-stimulating factor induces the release in the bone marrow of proteases that cleave c-KIT receptor (CD117) from the surface of hematopoietic progenitor cells.. Exp Hematol.

[18] Pelus LM, Bian H, King AG, Fukuda S (2004). Neutrophil-derived MMP-9 mediates synergistic mobilization of hematopoietic stem and progenitor cells by the combination of G-CSF and the chemokines GROβ/CXCL2 and GROβT/CXCL2Δ4.. Blood.

[19] Pruijt JF, Fibbe WE, Laterveer L, Pieters RA, Lindley IJ (1999). Prevention of interleukin-8-induced mobilization of hematopoietic progenitor cells in rhesus monkeys by inhibitory antibodies against the metalloproteinase gelatinase B (MMP-9).. Proc Natl Acad Sci USA.

[20] McQuibban GA, Butler GS, Gong JH, Bendall L, Power C (2001). Matrix metalloproteinase activity inactivates the CXC chemokine stromal cell-derived factor-1.. J Biol Chem.

[21] Heissig B, Hattori K, Dias S, Friedrich M, Ferris B (2002). Recruitment of stem and progenitor cells from the bone marrow niche requires MMP-9 mediated release of kit-ligand.. Cell.

[22] Kollet O, Dar A, Shivtiel S, Kalinkovich A, Lapid K (2006). Osteoclasts degrade endosteal components and promote mobilization of hematopoietic progenitor cells.. Nat Med.

[23] Winkler IG, Hendy J, Coughlin P, Horvath A, Lévesque JP (2005). Serine protease inhibitors serpina1 and serpina3 are down-regulated in bone marrow during hematopoietic progenitor mobilization.. J Exp Med.

[24] Kuiperij HB, van Pel M, de Rooij KE, Hoeben RC, Fibbe WE (2009). Serpina1 (alpha1-AT) is synthesized in the osteoblastic stem cell niche.. Exp Heamtol.

[25] van Pel M, van Os R, Velders GA, Hagoort H, Heegaard PM (2006). Serpina1 is a potent inhibitor of IL-8-induced hematopoietic stem cell mobilization.. Proc Natl Acad Sci USA.

[26] Gasson JC, Golde DW, Kaufman SE, Westbrook CA, Hewick RM (1985). Molecular characterization and expression of the gene encoding human erythroid-potentiating activity.. Nature.

[27] Hayakawa T, Yamashita K, Tanzawa K, Uchijima E, Iwata K (1992). Growth-promoting activity of tissue inhibitor of metalloproteinases-1 (TIMP-1) for a wide range of cells. A possible new growth factor in serum.. FEBS Lett.

[28] Hayakawa T, Yamashita K, Ohuchi E, Shinagawa A (1994). Cell growth-promoting activity of tissue inhibitor of metalloproteinases-2 (TIMP-2).. J Cell Sci.

[29] Haviernik P, Diaz MT, Haviernikova E, Tse W, Stetler-Stevenson WG (2008). Hematopoiesis in mice is extremely resilient to wide variation in TIMP/MMP balance.. Blood Cells Mol Dis.

[30] Pavloff N, Staskus PW, Kishnani NS, Hawkes SP (1992). A new inhibitor of metalloproteinases from chicken: ChIMP-3. A third member of the TIMP family.. J Biol Chem.

[31] Yu WH, Yu S, Meng Q, Brew K, Woessner JF (2000). TIMP-3 binds to sulfated glycosaminoglycans of the extracellular matrix.. J Biol Chem.

[32] Lee MH, Atkinson S, Murphy G (2007). Identification of the extracellular matrix (ECM) binding motifs of tissue inhibitor of metalloproteinases (TIMP)-3 and effective transfer to TIMP-1.. J Biol Chem.

[33] Amour A, Slocombe PM, Webster A, Butler M, Knight CG (1998). TNF-alpha converting enzyme (TACE) is inhibited by TIMP-3.. FEBS Lett.

[34] Qi JH, Ebrahem Q, Moore N, Murphy G, Claesson-Welsh L (2003). A novel function for tissue inhibitor of metalloproteinases-3 (TIMP3): inhibition of angiogenesis by blockage of VEGF binding to VEGF receptor-2.. Nat Med.

[35] Nakajima H, Ito M, Smookler DS, Shibata F, Fukuchi Y (2010). TIMP-3 recruits quiescent hematopoietic stem cells into active cell cycle and expands multipotent progenitor pool.. Blood.

[36] Apte SS, Olsen BR, Murphy G (1995). The gene structure of tissue inhibitor of metalloproteinases (TIMP)-3 and its inhibitory activities define the distinct TIMP gene family.. J Biol Chem.

[37] Hawkes SP, Li H, Taniguchi GT (2001). Zymography and reverse zymography for detecting MMPs, and TIMPs.. Methods Mol Biol.

[38] Kamei M, Hollyfield JG (1999). TIMP-3 in Bruch's membrane: changes during aging and in age-related macular degeneration.. Invest Ophthalmol Vis Sci.

[39] Lundberg P, Allison SJ, Lee NJ, Baldock PA, Brouard N (2007). Greater bone formation of Y2 knockout mice is associated with Increased osteoprogenitor numbers and altered Y1 receptor expression.. J Biol Chem.

[40] Short BJ, Brouard N, Simmons PJ (2009). Prospective isolation of mesenchymal stem cells from mouse compact bone.. Methods Mol Biol.

[41] Winkler IG, Sims NA, Pettit AR, Barbier V, Nowlan B (2010). Bone marrow macrophages maintain hematopoietic stem cell (HSC) niches and their depletion mobilizes HSC.. Blood.

[42] Robbins PB, Yu XJ, Skelton DM, Pepper KA, Wasserman RM (1997). Increased probability of expression from modified retroviral vectors in embryonal stem cells and embryonal carcinoma cells.. J Virol.

[43] Zeng Y, Rosborough RC, Li Y, Gupta AR, Bennett J (1998). Temporal and spatial regulation of gene expression mediated by the promoter for the human tissue inhibitor of metalloproteinases-3 (TIMP-3)-encoding gene.. Dev Dyn.

[44] Calvi LM, Adams GB, Weibrecht KW, Weber JM, Olson DP (2003). Osteoblastic cells regulate the haematopoietic stem cell niche.. Nature.

[45] Visnjic D, Kalajzic Z, Rowe DW, Katavic V, Lorenzo J (2004). Hematopoiesis is severely altered in mice with an induced osteoblast deficiency.. Blood.

[46] Raaijmakers MH, Mukherjee S, Guo S, Zhang S, Kobayashi T (2010). Bone progenitor dysfunction induces myelodysplasia and secondary leukaemia.. Nature.

[47] Mendez-Ferrer S, Michurina TV, Ferraro F, Mazloom AR, MacArthur BD (2010). Mesenchymal and haematopoietic stem cells form a unique bone marrow niche.. Nature.

[48] Marquez-Curtis LA, Dobrowsky A, Montano J, Turner AR, Ratajczak J (2001). Matrix metalloproteinase and tissue inhibitors of metalloproteinase secretion by haematopoietic and stromal precursors and their production in normal and leukaemic long-term marrow cultures.. Br J Haematol.

[49] Villeneuve J, Block A, Le Bousse-Kerdiles MC, Lepreux S, Nurden P (2009). Tissue inhibitors of matrix metalloproteinases in platelets and megakaryocytes: a novel organization for these secreted proteins.. Exp Hematol.

[50] Lowry WE, Blanpain C, Nowak JA, Guasch G, Lewis L (2005). Defining the impact of beta-catenin/Tcf transactivation on epithelial stem cells.. Genes & development.

[51] Anand-Apte B, Bao L, Smith R, Iwata K, Olsen BR (1996). A review of tissue inhibitor of metalloproteinases-3 (TIMP-3) and experimental analysis of its effect on primary tumor growth.. Biochem Cell Biol.

[52] Gomez DE, Alonso DF, Yoshiji H, Thorgeirsson UP (1997). Tissue inhibitors of metalloproteinases: structure, regulation and biological functions.. Eur J Cell Biol.

[53] Blavier L, Delaisse JM (1995). Matrix metalloproteinases are obligatory for the migration of preosteoclasts to the developing marrow cavity of primitive long bones.. J Cell Sci.

[54] Engsig MT, Chen QJ, Vu TH, Pedersen AC, Therkidsen B (2000). Matrix metalloproteinase 9 and vascular endothelial growth factor are essential for osteoclast recruitment into developing long bones.. J Cell Biol.

[55] Sternlicht MD, Werb Z (2001). How matrix metalloproteinases regulate cell behavior.. Ann Rev Cell Developmental Biol.

[56] Stickens D, Behonick DJ, Ortega N, Heyer B, Hartenstein B (2004). Altered endochondral bone development in matrix metalloproteinase 13-deficient mice.. Development.

[57] Inada M, Wang Y, Byrne MH, Rahman MU, Miyaura C (2004). Critical roles for collagenase-3 (Mmp13) in development of growth plate cartilage and in endochondral ossification.. Proc Natl Acad Sci.

[58] Geoffroy V, Marty-Morieux C, Le Goupil N, Clement-Lacroix P, Terraz C (2004). In vivo inhibition of osteoblastic metalloproteinases leads to increased trabecular bone mass.. J Bone Miner Res.

[59] Schiltz C, Marty C, de Vernejoul MC, Geoffroy V (2008). Inhibition of osteoblastic metalloproteinases in mice prevents bone loss induced by oestrogen deficiency.. J Cell Biochem.

[60] Sato T, Foged NT, Delaisse JM (1998). The migration of purified osteoclasts through collagen is inhibited by matrix metalloproteinase inhibitors.. J Bone Miner Res.

[61] Bian J, Wang Y, Smith MR, Kim H, Jacobs C (1996). Suppression of in vivo tumor growth and induction of suspension cell death by tissue inhibitor of metalloproteinases (TIMP)-3.. Carcinogenesis.

[62] Anand-Apte B, Pepper MS, Voest E, Montesano R, Olsen B (1997). Inhibition of angiogenesis by tissue inhibitor of metalloproteinase-3.. Invest Ophthalmol Vis Sci.

[63] Mahller YY, Vaikunth SS, Ripberger MC, Baird WH, Saeki Y (2008). Tissue inhibitor of metalloproteinase-3 via oncolytic herpesvirus inhibits tumor growth and vascular progenitors.. Cancer Res.

[64] Niida S, Kaku M, Amano H, Yoshida H, Kataoka H (1999). Vascular endothelial growth factor can substitute for macrophage colony-stimulating factor in the support of osteoclastic bone resorption.. J Exp Med.

[65] Mahmoodi M, Sahebjam S, Smookler D, Khokha R, Mort JS (2005). Lack of tissue inhibitor of metalloproteinases-3 results in an enhanced inflammatory response in antigen-induced arthritis.. Am J Pathol.

[66] Smookler DS, Mohammed FF, Kassiri Z, Duncan GS, Mak TW (2006). Cutting edge: tissue inhibitor of metalloproteinase 3 regulates TNF-dependent systemic inflammation.. J Immunol.

[67] Horiuchi K, Kimura T, Miyamoto T, Miyamoto K, Akiyama H (2009). Conditional inactivation of TACE by a Sox9 promoter leads to osteoporosis and increased granulopoiesis via dysregulation of IL-17 and G-CSF.. J Immunol.

[68] Althoff K, Reddy P, Voltz N, Rose-John S, Mullberg J (2000). Shedding of interleukin-6 receptor and tumor necrosis factor alpha. Contribution of the stalk sequence to the cleavage pattern of transmembrane proteins.. Eur J Biochem.

[69] Brou C, Logeat F, Gupta N, Bessia C, LeBail O (2000). A novel proteolytic cleavage involved in Notch signaling: the role of the disintegrin-metalloprotease TACE.. Mol Cell.

[70] Six E, Ndiaye D, Laabi Y, Brou C, Gupta-Rossi N (2003). The Notch ligand Delta1 is sequentially cleaved by an ADAM protease and gamma-secretase.. Proc Natl Acad Sci.

[71] Rovida E, Paccagnini A, Del Rosso M, Peschon J, Dello Sbarba P (2001). TNF-alpha-converting enzyme cleaves the macrophage colony-stimulating factor receptor in macrophages undergoing activation.. J Immunol.

[72] Garton KJ, Gough PJ, Philalay J, Wille PT, Blobel CP (2003). Stimulated shedding of vascular cell adhesion molecule 1 (VCAM-1) is mediated by tumor necrosis factor-alpha-converting enzyme (ADAM 17).. J Biol Chem.

[73] Cruz AC, Frank BT, Edwards ST, Dazin PF, Peschon JJ (2004). Tumor necrosis factor-alpha-converting enzyme controls surface expression of c-Kit and survival of embryonic stem cell-derived mast cells.. J Biol Chem.

[74] Horiuchi K, Morioka H, Takaishi H, Akiyama H, Blobel CP (2009). Ectodomain shedding of FLT3 ligand is mediated by TNF-alpha converting enzyme.. J Immunol.

[75] Akashi K, Traver D, Miyamoto T, Weissman IL (2000). A clonogenic common myeloid progenitor that gives rise to all myeloid lineages.. Nature.

[76] Semerad CL, Christopher MJ, Liu F, Short B, Simmons PJ (2005). G-CSF potently inhibits osteoblast activity and CXCL12 mRNA expression in the bone marrow.. Blood.

[77] Lundberg P, Allison SJ, Lee NJ, Baldock PA, Brouard N (2007). Greater bone formation of Y2 knockout mice is associated with increased osteoprogenitor numbers and altered Y1 receptor expression.. J Biol Chem.

[78] Winkler IG, Barbier V, Wadley R, Zannettino A, Williams S (2010). Positioning of bone marrow hematopoietic and stromal cells relative to blood flow in vivo: Serially reconstituting hematopoietic stem cells reside in distinct non-perfused niches.. Blood.

[79] Sims NA, Brennan K, Spaliviero J, Handelsman DJ, Seibel MJ (2006). Perinatal testosterone surge is required for normal adult bone size but not for normal bone remodeling.. Am J Physiol Endocrinol Metab.

