# Supplementary figures and images for: Tissue Inhibitor of Metalloproteinase-3 (TIMP-3) Regulates Hematopoiesis and Bone Formation In Vivo
Source: PLoS One. 2010 Sep 30;5(9):e13086. doi: 10.1371/journal.pone.0013086 (PMC2948005; doi:10.1371/journal.pone.0013086)

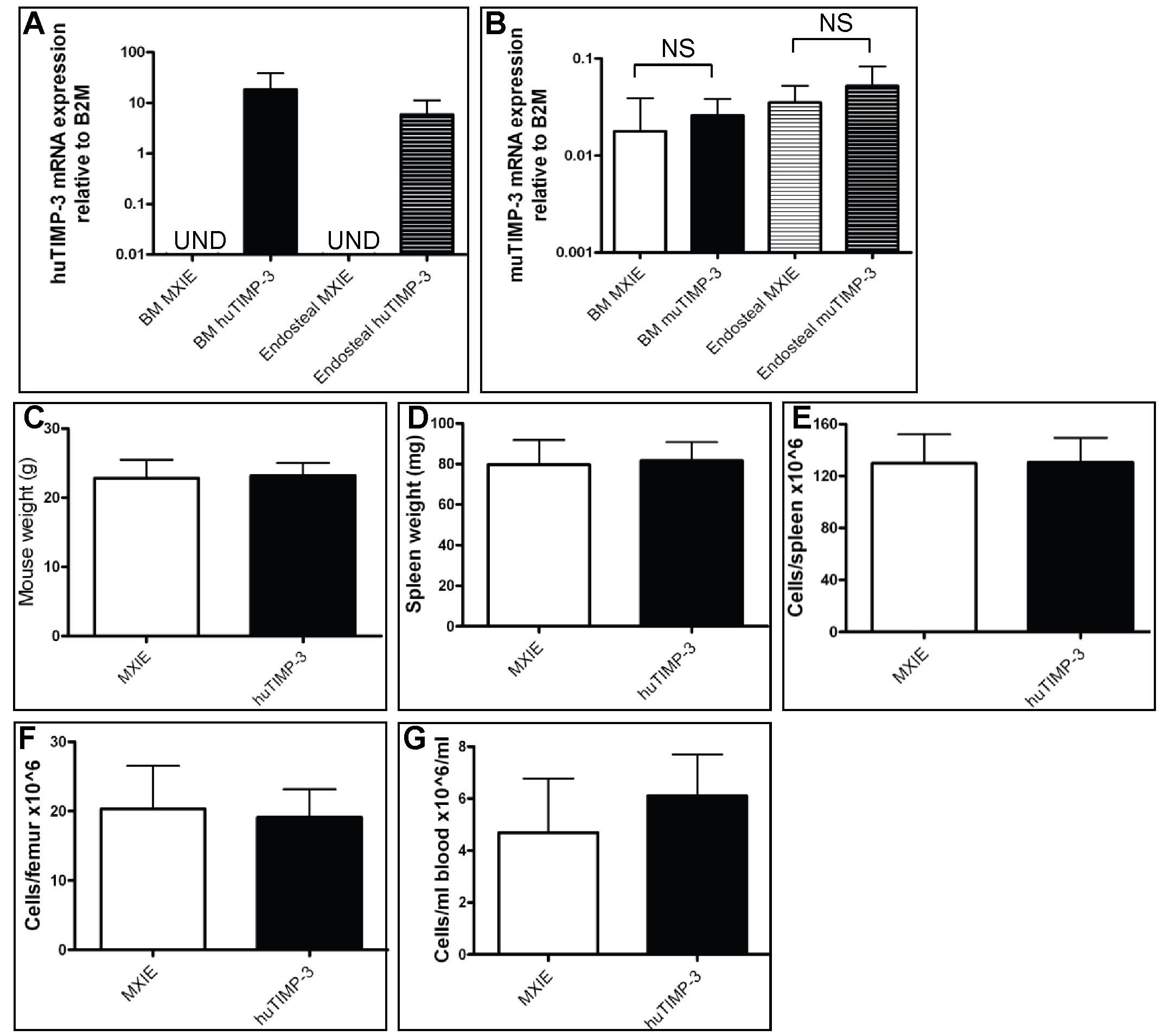

Supplement: Figure S1 — Lack of effect of huTIMP-3 overexpression in hematopoietic cells on mouse weight and white blood cell counts in blood, BM and spleen 14 weeks post-transplant. RT-qPCR for huTIMP-3 expression (A) and endogenous muTIMP-3 expression (B) in central BM cells and endosteal cells from mice transplanted with HSC transduced with empty MXIE vector or MXIE-huTIMP3 vector. Data are normalized to B2M mRNA and are mean±SD of 5 mice per group. UND is for undetected. Body weight (C), spleen weight (D), white cell counts in spleen (E), femur (F) and blood (G). Data are mean±SD of 9 mice per group. (0.77 MB TIF) [file pone.0013086.s001.tif]
